#  The Role of p66shc in Oxidative Stress and Apoptosis 

**Published:** 2010

**Authors:** E.R. Galimov

**Affiliations:** Belozersky Institute of Physico-Chemical Biology, Moscow State University

**Keywords:** apoptosis, reactive oxygen species, p66shc, mitochondria

## Abstract

*p66shc*is a gene that regulates the level of reactive oxygen species (ROS), apoptosis induction, and lifespan in mammals. Mice**knocked out for*p66shc*have a lifespan*~*30% longer**and**demonstrate an enhanced resistance to oxidative stress and age-related pathologies such as hypercholesterolemia, ischemia, and hyperglycemia. In this respect, p66shc is a promising pharmacological target for the treatment of age-related diseases. In this review, an attempt has been made to survey and put to a critical analysis data concerning the involvement of p66shс in the different signaling pathways that regulate oxidative stress and apoptosis.

##  Introduction 

The identification of the mutations that lead to the prolongation of the lifespan of various model organisms shows that aging can be considered as a genetic program [1]. One of these genes is *p66shc* , the deletion of which results in a 30% increase in the lifespan. It is important to note that miceknocked out for *p66shc* , in comparison with other mouse models with a prolonged lifespan (e.g., mice with the deleted gene of a growth hormone receptor), are fertile and exhibit a normal phenotype [2]. These mice are resistant to oxidative stress and age-related pathologies such as atherosclerosis [3], endothelial disorders [4], AGE (advanced glycation end products)-dependent glomerulopathy related to diabetes mellitus [5, 6], and ethanol-induced liver affection [7].

P66shc is an adaptor protein which is coded for by a single locus in *Drosophila (dShc)* , and by four loci in mammals – *Shc* ( *ShcA* ), *Sli* ( *ShcB* ), *Rai* ( *ShcC* ) [8], and *RalP* [9]. The four mammalian loci code for at least 7 proteins due to the usage of alternative start codons and alternative splicing. Three isoforms encoded by the *ShcA* locus are designated according to their molecular weights as p46shc, p52shc, and p66shc, respectively. These proteins participate in the regulation of proliferation (p46shc and p52shc) and apoptosis (p66shc) [8].

**Fig. 1:**
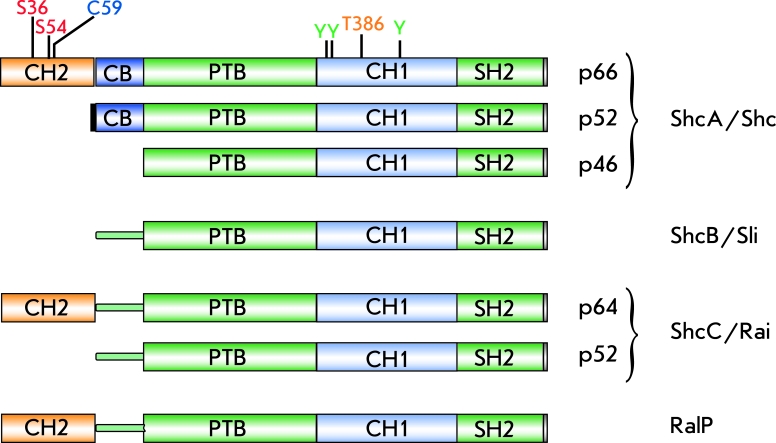
Schematic presentation of domain structure of SHC-like proteins. Modified amino acid residues denoted as: serine 36 and 54 (S36 and S54), cysteine 59 (C59), threonine 386 (T386) and tyrosines (Y) phosphorylated during signal transductionfromtyrosine kinase receptors.

P66shc is considered to be a relatively “young” protein since it is not found in yeast ( *Saccharomyces* ), nematodes ( *Caenorhabditis* ), and insects ( *Drosophila* ) but appears in amphibians ( *Xenopus* ), fishes ( *Fugu rubripes* ), and mammals [8]. Since it is the longest isoform, p66shc contains all the most ancient domains that are found in short isoforms p46shc and p52shc (Fig. 1), including: the N-terminal phosphotyrosine-binding domain (PTB), the middle collagen homology domain (CH1), and the C-terminal Src-homology domain (SH2). The isoforms that are longer than p46shc contain some additional N-terminal domains: the cytochrome c binding domain (CB), which is common to both p52shc and p66shc, and the collagen-homology domain (CH2), which is unique to p66.

P46shc and p52shc fulfill the function of adaptor proteins transmitting signals from various tyrosine-kinase receptors, which phosphorylate tyrosine residues in these proteins. Phosphorylation of p46shc/p52shc induces the formation of a complex between GRb2 (adaptor protein) and SOS (nucleotide exchange factor), which leads to RAS activation and the induction of the mitogene-activated protein kinases (MAPK) pathway. Although p66shc as p46shc/p52shc is phosphorylated by tyrosine-kinase receptors and interact with the GRb2/SOS complex, it, apparently, does not activate MAPK [10]. The competition between p66shc and p52shc for binding with GRb2 [11] and p66shc-induced displacement of SOS from its complex with GRb2 [12, 13] can mediate the regulation of signal transduction from receptors. However, experiments on animal models knocked out for *p66shc * showed that the role of this protein in lifespan regulation, oxidative stress, and apoptosis is accounted for by the CH2 and CB domains, which are not essential for the primary adaptor function of the protein.

##  P66, Oxidative Stress, and Apoptosis. 

Studies on mice with a knocked out *p66shc * gene revealed decreased levels of intracellular ROS, as determined by means of ROS-sensitive probes, as well as reduced levels of oxidative damages to DNA and proteins, estimated by measuring 8-oxo-deoxiguanosine and nitrotyrosines [3,4,14-16]. The mutant mice exhibited higher resistance to paraquat-induced oxidative stress [2]. Studies on various *p66shc* -deficient cell lines (ones with a deleted *p66shc* -gene, or cells with a dominant-negative phenotype caused by a Ser36Ala substitution in the target gene) derived from mice, rats, and a human showed that p66shc plays an important role in apoptosis induced by various agents (Table 1). P66shc-mediated oxidative stress is assumed to be the key factor in these experimental models of apoptosis. In particular, certain published data indicate the importance of p66shc-induced oxidative stress in the p53-dependent apoptotic pathway [14]. Data derived from physiological experiments have led to similar conclusions (Table 2).

Posttranslational modifications have been shown to play an important role in the pro-apoptotic activity of p66shc. For instance, phosphorylation at Ser36 located in the CH2-domain is indispensable in hydrogen peroxide- or UV-induced apoptosis [2]. Phosphorylation itself is carried out by such kinases as JNK (in response to UV or amyloid β-peptide) [23, 33], ERK [34], and PKCβ in response to treatment by H _2_ O _2_ [35]. Phosphorylation at Ser36 promotes interaction with 14-3-3 proteins [36], tyrosine phosphatase PTP-PEST [37], and prolylisomerase Pin-1 [35]. While the significance of the first two interactions remains unclear, Pin-1-mediated p66shc isomerization plays a crucial role in the regulation of p66shc transport into mitochondria and activation of mitochondrion-dependent apoptosis (see below).

However, despite the great amount of research confirming the pro-apoptotic functions of p66shc, some studies suggest that it can also exhibit antiapoptotic effects. In a human breast cancer model, as well as in a human stem cell model, it was shown that the suppression of *p66shc* expression protects from hypoxia-induced cytotoxicity. It was also found that low oxygen concentrations lead to p66shc activation, which, in turn, induces expression of the *Notch-3* gene. The latter accounts for the self-renewal of stem cells and their survival under hypoxia. Notch-3 induces the expression of carboanhydrase IX, which also accounts for a hypoxia resistant phenotype [38]. Therefore, a connection exists between p66shc and the pathway that underlies the protection of stem cells from hypoxia. These data clarify the role of p66shc in homeostasis and the self-renewal of stem cells, which are known to occupy specialized tissue compartments or niches containing small amounts of blood vessels and, hence, low oxygen concentrations. It is interesting to note that since the small GTPase Rac-1 is a well-known activator of p66-induced oxidative stress [25], it also plays an essential part in the maintenance and self-renewal of epidermal stem cells. Taken together, these data show that p66shc has a more complex role, acting as a “double-edged sword” in the regulation of apoptosis, based on environmental conditions and genetic context.

##  Mechanisms of P66-dependent Increase in Cellular ROS Levels 

 By now, we know the mechanisms p66shc exploits to increase intracellular ROS levels: activation of membrane-bound NADPH-oxidases, down-regulation of antioxidant enzymes synthesis, and generation of ROS in mitochondria. 

**p66-dependent Activation of Membrane-bound NADPH-oxidases**

**Fig. 2:**
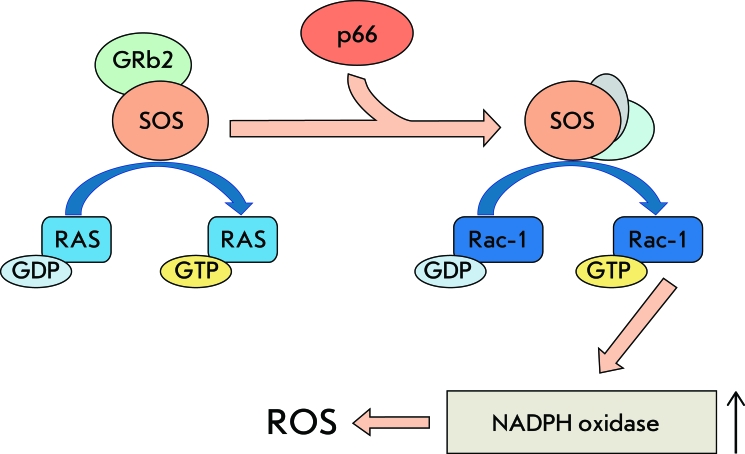
P66-dependent activation of plasma membrane NADPH oxidases. P66shc displaces nucleotide exchange factor SOS from Grb2 complex and promotes activation of small GTPase Rac1. Activated Rac1 stimulates NADPHoxidase complex formation and ROS generation.

First of all, it should be noted that p66shc can cause oxidative stress in the cell carrying out its primary function of adaptor protein. As was mentioned above, this protein can negatively regulate RAS-activation by means of displacing the nucleotide exchange factor SOS from its complex with GRb2. It has turned out that this chain of events can be accompanied by the SOS-dependent activation of small GTPase Rac-1, which consequently promotes the assembly of membrane-bound NADPH-oxidases and the production of ROS [12] (Fig. 2). It has been shown that p66shc is necessary for apoptosis induced by a constitutively active Rac-1 mutant. However, this apoptosis scenario does not involve the phosphorylation of Ser36 but phosphorylation of Ser54 and Thr386, instead, which promotes Rac-1-dependent stabilization of p66shc and protects against ubiquitin-mediated degradation [25]. In macrophages, where NADPH-oxidase serves as the major source of ROS, knockout of the *p66shc* gene undermines the formation of the active NADPH-oxidase complex, ultimately leading to a 40% decrease in ROS-formation.

**Table 1:** Cellular models of p66shc inactivation (via either *p66shc* gene deletion or expression of а dominant-negative mutant of p66shc at S36) with phenotype of apoptosis resistance

Cells	Cell line name	Organism	Apoptosis inducer	References
Embryonic fibroblasts	MEF	mouse	H_2_O_2_, UV, staurosporine, isothiocyanate, chloroform	[2, 15, 17, 18]
Primary cardiomyocyte		mouse	angiotensin II	[19]
Transformed renal epithelial cells	TKPTS	mouse	H_2_O_2_, cisplatin	[13]
Hepatocytes transgenic for human TGFa	AML12	mouse	hypoxia-reoxygenation	[20]
Endothelial progenitor cells	BM c-kit+	mouse	high glucose in media	[21]
Osteoblastic cells	OB-6, UAMS-32	mouse	H_2_O_2_	[22]
Pheochromocytoma	PC12	rat	beta amyloid, constitutively active Rac1 mutant	[23]
Cardiomyocyte	ARVM	rat	high glucose in media	[24]
Transformed fibroblast-like cells	COS7	green monkey	constitutively active Rac1 mutant	[25]
Neuroblastoma	SH-SY5Y	human	beta amyloid	[23]
Human podocytes immortalized with SV40-T-antigen	CIDHPs	human	HIV-1 transfection	[26]
Prostatic carcinoma	PC3, LNCaP	human	isothiocyanate	[18]
Cervical carcinoma	HeLa	human	H_2_O_2_	[27]
Osteosarcoma	SaOs-2	human	H_2_O_2_	[27]
Retinal pigmented epithelial Cells	RPE	human	H_2_O_2_	[28]
Lymphoma	Jurcat	human	hypoxia, calcium ionophores	[29]
Transformed renal epithelial cells	φNx-293	human	cell detachment from a solid matrix	[30]
Endothelial cells	HuVec	human	cell detachment from a solid matrix	[30]

**Table 2:** p66 involvement in the development of pathologies associated with oxidative stress and demonstrated on physiological models (experiments with *p66shc * knockout animals or comparison between young and elderly individuals)

Pathology associated with apoptosis	Organism and genetic line	Reference
Experimental diabetic glomerulopathy	mouse, SV/129	[5]
Vascular cell apoptosis and atherogenesis induced by high-fat diet	mouse, SV/129	[3]
Cardiomyocyte apoptosis in experimental model of diabetes induced by streptozotocin	mouse, SV/129	[31]
Cerebral cortex hypoxia	rat, Sprague–Dawley	[32]

**p66shc and Regulation of Expression of Antioxidant Enzymes**

**Fig. 3:**
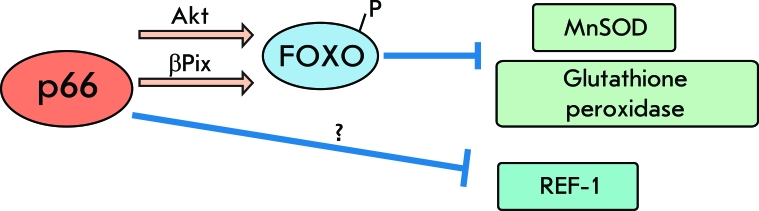
P66 role in regulation of the cellular antioxidant defense system. P66 can downregulate antioxidative ferments and regulatory factors. This downregulation can be induced through both Akt-dependent and Akt-independent inactivation of Forkhead transcription factors.

Previous research has shown that p66shc reduces the expression of such antioxidant enzymes and regulatory factors as glutathione peroxidase-1 [28], MnSOD [7, 20, 28], and REF-1 [20] by means of down-regulation of Forkhead-type transcription factors (e.g., Foxo3a) [23, 40, 41]. In the course of oxidative stress, serine/threonine protein kinase Akt undergoes phosphorylation and in turn phosphorylates and inactivates Foxo3a. This reaction requires the presence of p66shc in the cell [40] and also its phosphorylation at Ser36 [42]. Outside of that, some data suggest that p66shc, in complex with βPix (a nucleotide exchange factor for Rac-1 and Cdc42), can cause Akt-independent phosphorylation and the inactivation of Foxo3a [43] (Fig. 3).

It should be mentioned that these data are in disagreement with a number of publications which argue that p66shc does not affect the levels of antioxidant enzymes [4, 15-17].

**p66shc and Mitochondria-mediated Apoptosis**

The fact that cells carrying deletion of the *p66shc* gene are resistant to various inductors of mitochondria-mediated apoptosis implies a direct interaction between p66 and mitochondria.

*Mitochondrial localization of p66shc and its transport to mitochondria. * Studies on the cellular localization of p66shc showed that 32% of this protein is localized in the cytoplasm; 24% is in the endoplasmic reticulum; and 44%, in mitochondria [17]. Inside the mitochondria, p66shc is distributed in the following manner: 35% is in the intramembrane space, 56% is associated with the inner membrane, and 9% is located in the mitochondrial matrix [15]. According to other data, mitochondria contain only 10% of cellular p66shc [44]. These differences told arise from the changes in p66shc intracellular localization caused by external influences.

The stimuli that promote p66shc translocation into mitochondria are usually pro-apoptotic factors, such as UV radiation and treatment with H _2_ O _2 _ [17, 35]. However, the mechanism of p66shc transport into mitochondria remains unknown. It is known that a short Shc isoform p46shc is also localized in mitochondria and contains the signal of mitochondrial import [45]. However, mutations in a similar mitochondrial import sequence in p66 did not affect its localization - which probably means that this signal is somehow masked by the N-terminal CH2-domain [44]. It was also shown that p66shc is associated with protein complexes that contain mHSP70, TIM, and TOM subunits and mediate protein transport into mitochondria. P66 is thought to be inactive in these complexes; however, it is believed that under oxidative stress p66 dissociates and thus acquires active conformation [46].

**Fig. 4:**
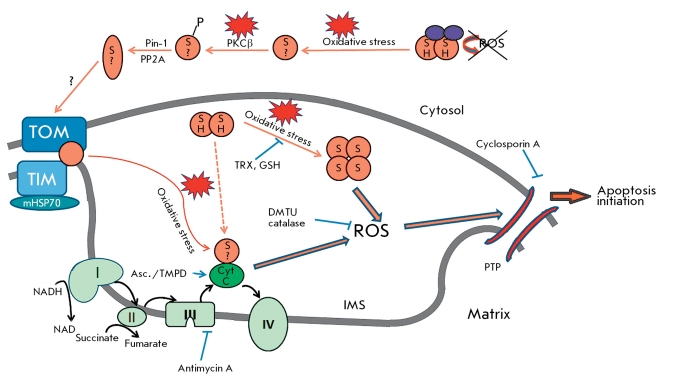
Model for induction proapoptotic signaling in mitochondria. p66shc (denoted as orange circle) can exist as dimer (circles where “SH” indicates reduced cysteine residue 59) and tetramer (circles with “S” indicating an oxidized cysteine residue 59). Circles with “S?” indicating unknown redox state of a cysteine residue 59 that is responsible for tetramerisation p66. Interaction of p66shc with peroxiredoxin 1 (Prx1, denoted as violet oval) in cytosol results in a complex presumably consisting of dimeric p66 and dimeric Prx1 in which Prx1 peroxidase activity degrades p66-generated ROS. Oxidative stress leads to dissociation of the complex; PKCβ phosphorylates released p66 on S36. Phosphorylated p66 becomes a target for prolyl isomerase Pin1, which recognizes proline following phosphorylated serine residue. After izomerization PP2A dephosphorylates p66shc, which is then transported into mitochondria. During oxidative stress p66shc is released from the high *-* molecular *-* mass complex that containsTOM, TIM, and mHsp70. Released p66shc acts as oxidoreductase and transfers electrons from reduced Cyt C to oxygen. As a result, generated ROS lead to permeability-transition pore (PTP) opening and induction of apoptosis. These consequences may appear after tetramericp66formation duringoxidative stress and following depletion of the thoredoxin and glutathione pool (for full explanation, see text). Asc. – ascorbate *;* DMTU – dimethylthiourea *; * GSH – glutathione; IMS – intermembrane space of mitochondria *;* PTP – permeability transition pore *;* TMPD – N,N,N’,N’- tetramethyl-p-phenyldiamine; TRX – thioredoxin.

According to data presented in [35], the signal pathway that initiates p66shc translocation into mitochondria upon H _2_ O _2 _ treatment includes the activation of PKCβ, which phosphorylates p66 at Ser36. Phosphorylated p66 becomes a target of prolylpolymerase Pin-1 that recognizes a proline residue, which follows phosphorylated serine. After isomerization, p66shc is dephosphorylated by PP2A phosphatase and transported into mitochondria. The latter event is confirmed by the fact that the pool of mitochondrial p66shc is not phosphorylated [15] (Fig. 4). It was also shown in [35] that the absence of p66shc also results in altered Ca-signaling and prevents the fragmentation of mitochondria, which is related to resistance to apoptosis.

*Redox proapoptotic activity of p66shc and the production of ROS. * Current views hold that, in the course of mitochondrial-dependent apoptosis, various signals (ROS, elevated Ca, uncoupled oxidative phosphorylation) promote the formation of a multi-subunit protein complex, which eventually forms a pore in the inner mitochondrial membrane. The permeabilization of both mitochondrial membranes results in the release of Cyt C and other proteins in the cytoplasm, apoptosome formation, and caspase activation [47].

Pelicci *et al* . have suggested a mechanism explaining the role of p66shc in mitochondrium-dependent apoptosis [15]. It turns out that p66shc is necessary for a drop in the membrane’s potential and Cyt C release in cytosol. Moreover, the addition of cyclosporine A, an inhibitor of permeability transition pore formation, blocked the pro-apoptotic function of p66shc [17]. *In vitro* showed that the addition of recombinant p66shc to isolated mitochondria with a permeabilized outer membrane results in mitochondria swelling as a result of permeability transition pore formation. This effect was also inhibited by cyclosporine A, catalase, antioxidant dimethylthiourea, and the inhibitor of complex III of the respiratory chain antimycin A, as well as being dependent on respiratory substrates. Therefore, the obtained data is evidence that permeability-transition pore formation is a key step in p66-mediated apoptosis and that ROS and respiration are also necessary in this process.

The application of electrochemical analysis and fluorescent redox-sensitive probes revealed that p66 acts as a redox enzyme and transfers electrons from reduced Cyt C to oxygen. The interaction of p66 with Cyt C is mediated by the CB-domain, which was confirmed by ELISA and site-directed mutagenesis [17]. Incomplete oxygen reduction leads to ROS production, which in turn promotes the formation of a permeability-transition pore. In confirmation of this hypothesis, ROS formation was shown upon the addition of p66 to mitochondria in the absence of substrates of respiration in a medium supplemented with ascorbate/TMPD., a redox couple that selectively reduces Cyt C. ROS formation was also observed even in the absence of mitochondria upon mixing of Cyt C and p66shc. However, in this case, the presence of copper ions was indispensable.

Therefore, the Pelicci group suggested the following mechanism of pro-apoptotic action of p66shc (Fig. 4): under normal conditions, p66shc is inactivated and represents a part of the multi-subunit complex comprising TIM, TOM, and mHSP70. Oxidative stress causes p66shc to dissociate from the complex. As a result, p66shc acts as a redox enzyme and transfers electrons from the reduced Cyt C to oxygen. The incomplete reduction of oxygen results in ROS production, which promotes the formation of a permeability-transition pore, mitochondrial swelling, Cyt C release to cytosol, apoptosome assembly, and caspase activation.

It is important to realize that the suggested mechanism of p66shc redox activity, which includes Cyt C as an electron donor, is only a reality *in vitro* . In order to validate this hypothesis, one needs to carry out additional experiments based on fluorescent probes for measuring ROS levels in the mitochondria of cells with an inactive respiratory chain (Rho-0 cells), as well as in cells devoid of *Cyt C* .

It should be noted that the described model has many weak points. For example, p66shc does not contain any well-known redox domains or metal-binding domains. Therefore, its ability to generate ROS only in the presence of copper ions can be an artifact, since copper ions are known to be able to generate ROS during Fenton’s reaction, which could affect the results obtained by means of redox-sensitive fluorescent probes. It is also known that Cyt C, upon oligomerization, can produce ROS as a result of auto-oxidation [48-52]. Taking this into account, one can suggest that p66shc, upon binding with Cyt C, can act as a factor promoting oligomerization and the auto-oxidation of Cyt C, similarly to ptothymosine α, but not as a redox enzyme [53].

*Studies on an isolated CH2-CB-domain* . The mechanism of action of p66shc was also studied in *in vitro* experiments with an isolated CH2-CB-domain. It turns out that the recombinant CH2-CB-domain can exist in two forms: reduced, as a dimer, and oxidized, as a tetramer (or a dimer of dimers) which contains disulfide bridges between residues Cys59. The addition of both the CH2-CB-domain and the full-length protein to mitochondria resulted in the formation of a permeability transition pore and swelling; however, only the tetramer exhibited such a pro-apoptotic activity. In contradiction to the data presented in [15], which suggested that ROS production is directly connected to permeability-transition pore formation, the tetramer form of the recombinant CH2-CB-domain generated less ROS than the dimer when mixed with isolated mitochondria.

 Apart from that, the CH2-CB-domain was shown to trigger ROS production in the presence of dithionite (as an electron donor) and copper ions. However, this effect was not reproduced when dithionite was replaced with Cyt C as an electron donor. Apparently, the other domains of p66shc are necessary either for interaction with Cyt C or for maintenance of the CH2-CB-domain in proper conformation. 

 As a result of the above-described findings, a refined model of p66-mediated apoptosis was suggested. The model assumes that under normal conditions the tetrameric form of p66 is reduced by mitochondrial antioxidant systems. Under stress conditions, however, antioxidant systems are not able to retain p66shc in their reduced dimeric state, so the oxidized tetrameric forms generate ROS locally, which triggers permeability-transition pore formation and apoptosis. 

 It is important to note that a direct connection between the ROS-generating and pro-apoptotic functions of p66shc does not necessarily exist. For example, a confirmation of this fact can be the lower level of ROS production in the case of pro-apoptotic tetrameric forms of p66shc in comparison to the dimer, which does not induce apoptosis. 

*p66 as a redox sensor* . Extensive experiments on the isolated CH2-CB-domain revealed a new interaction partner of p66shc – peroxiredoxine 1 (Prx1). Prx1 is a member of the peroxidase family. These enzymes regulate the redox balance in the cell. Prx1 can exist in the form of a dimer, a decamer (5 dimers) or a multimer. Prx1 is basically localized in cytosol, though recent proteomic studies have revealed its presence in the perimitochondrial compartment. Under normal conditions, Prx1 exists predominantly in dimeric form, which functions as peroxidase. When the cell is under stress, Prx1 undergoes oxidation and forms decamers that possess lower peroxidase activity. However, upon these transformations Prx1 acquires a chaperone function. Severe oxidative stress leads to the formation of the multimer, which is also devoid of peroxidase activity but can function as a chaperone [56-58].

Interactions between the CH2-CB-domain of p66shc and Prx1 result in the destabilization of the decameric form of Prx1 and favor dimeric form transition. In turn, Prx1, in complex with p66shc, retains p66shc in dimeric form by means of a disulfide exchange between the two proteins. The resulting hybrid complex consists of dimeric p66shc, which generates ROS but has low pro-apoptotic activity, and dimeric Prx1, which functions as a peroxidase. Thus, under normal conditions dimeric Prx1 stabilizes the inactive state of p66shc and degrades ROS which are produced by p66shc. Under stress conditions, cystein residues of proteins are oxidized, resulting in disassembly of the complex: so, the uncomplexed p66shc can now participate in apoptosis induction [55]. Therefore, the complex of dimeric p66shc and dimeric Prx1 can be considered as a sensor that detects the level of cellular ROS and induced apoptosis when excessive amounts of ROS are accumulated (Fig. 4).

##  Conclusion 

 According to current views, p66shc is a pro-apoptotic protein which regulates oxidative stress and induces the mitochondrial apoptosis pathway by means of redox activity. Although the physiological role of p66shc has been extensively studied, little is known about the precise mechanism of action underlying its redox functions and participation in apoptosis induction. Further studies will endeavor to elucidate the precise mechanism of p66shc translocation into mitochondria and the localization of p66shc-dependent ROS production in the cell. Although p66shc phosphorylation at Ser36 is one of the indispensible steps of apoptosis, it was shown that the mitochondrial pool of p66 is not phosphorylated. This indicates that p66shc phosphorylated at Ser36 probably participates in pro-apoptotic events outside mitochondria. 

 P66shc is undoubtedly interesting in terms of the investigation of oxidative stress, apoptosis, and the aging related to it. It is important to note that, to estimate the involvement of p66shc in age-related disorders, it is necessary to better understand its role in cancer. A deeper understanding of the regulatory pathways and structural and mechanistic bases of p66shc redox activity can be important for developing pharmacological approaches to the treatment of age-related disorders. 

## Abbreviations

Akt: protein kinase B
Cdc42 (cell division control protein 42 homolog): a small GTPase of the Rho-subfamily
Cyt C: cytochrome c
DMTU: dimethylthiourea
Grb2: growth factor receptor-bound protein 2
ERK: extracellular signal-regulated kinases
HIV-1: human immunodeficiency virus 1
IMS: intermembrane space of mitochondria
JNK: c-Jun N-terminal kinases
MAPK: mitogen-activated protein kinases
mHSP70: mitochondrial 70 kilodalton heat shock proteins
MnSOD: mitochondrial superoxide dismutase
PKCβ: protein kinase Cβ
Pin-1: peptidyl-prolyl cis/trans isomerase
PP2A: Protein phosphatase 2
Prx1: peroxiredoxin 1
PTP: permeability transition pore
PTP-PEST: protein tyrosine phosphatase that contains a C-terminal PEST motif
Rac-1: ras-related C3 botulinum toxin substrate 1
RAS: rat sarcoma viral oncogene
REF-1: redox factor 1
ROS: reactive oxygen species
SOS1 (Son of Sevenless): guanine nucleotide-binding protein
TIM: translocase of the inner membrane of mitochondria
TMPD: N,N,N’,N’- tetramethyl-p-phenyldiamine
TOM: translocase of the outer membrane of mitochondria
